# Social Isolation and the Use of Technology in Caregiving Dyads Living With Dementia During COVID-19 Restrictions

**DOI:** 10.3389/fpubh.2022.697496

**Published:** 2022-02-04

**Authors:** Viktoria Hoel, Karin Wolf-Ostermann, Eliva Atieno Ambugo

**Affiliations:** ^1^Department of Nursing Science Research, Institute for Public Health and Nursing Research, University of Bremen, Bremen, Germany; ^2^Leibniz ScienceCampus Digital Public Health, Bremen, Germany; ^3^Department of Health, Social and Welfare Studies, Faculty of Health and Social Sciences, University of South-Eastern Norway (USN), Kongsberg, Norway

**Keywords:** dementia, COVID-19 pandemic, dyadic relationships, caregiving, technology, social participation, isolation

## Abstract

**Background:**

People with dementia (PwD) and their informal caregivers (caregiving dyads) face multiple impacts of the COVID-19 pandemic, including restricted social support services and social isolation. With limited opportunities for caregiving dyads to participate in social activities during the pandemic, the potential of social technology to support social participation and dyadic relationships should be explored. As a part of an ongoing feasibility trial, this study assesses how COVID-19 has impacted community-dwelling dyads in a dementia caregiving context. The dyads' use of social technology and their motivations to invite technology into social interactions are explored.

**Methods:**

A pilot case study employing baseline interview data from three community-dwelling caregiving dyads. Each dyad consisted of a husband with a dementia diagnosis and his wife, who performed most caregiving tasks. Interviews were audio-recorded, transcribed verbatim, and subjected to inductive thematic analysis. Two researchers independently coded the data, and collated the codes and themes collaboratively.

**Results:**

Two themes and seven subthemes were identified: (i) living with dementia during COVID-19 (subthemes: social and leisure activities, dyadic interactions, adjusting as caregiver); and (ii) the role of technology in a pandemic (subthemes: facilitating social activities, facilitating dementia care-related activities, barriers and facilitators to using social technology, the underlying motivation to invite technology into interactions). Dyads who were socially active pre-COVID-19, and who managed to make good use of technology to facilitate and maintain their social engagement during COVID-19, reported to have been less negatively impacted by COVID-related social restrictions.

**Conclusion:**

The dyads differed in how COVID-19 restrictions impacted their lives and how they coped with dementia, revealing different motivations for wanting to invite technology into their social interactions. During and beyond this pandemic, social technology can be a valuable tool for promoting social participation in this population, especially when in-person social contact is restricted. Successful uptake of social technology is dependent on customizing it to the individual's needs and conditions. Therefore, efforts are needed to tackle barriers that exist for older adults in using such technology.

## Background

People with dementia (PwD) and their informal caregivers face multiple impacts of the ongoing COVID-19 pandemic. Firstly, as the majority of this patient group are older (1), they have a higher risk of illness and mortality (2–4). Secondly, the COVID-19 restrictions led to most non-essential services being closed, leaving many community-dwelling PwD and their informal caregivers with few activities to engage in outside their homes. However, social participation is recognized as influential for health and well-being (5–7). Being largely confined to their homes since March 2020 has led to isolation by restricting the support networks available to caregiving dyads. This, in turn, might adversely impact the coping capacity of informal caregivers, as perceived low levels of social support and feelings of loneliness are related to lower resilience (8–10). Informal caregivers of PwD have been found to be exposed to the greatest degree of stress and burden compared to caregiving groups of patients with other diagnostic conditions (3). Therefore, “caring for the carer” approaches are needed to meet not only the physical but also the emotional needs of informal caregivers through psychosocial interventions (11).

Facilitating opportunities for in-the-moment connections in daily life between PwD and informal caregivers is considered an important goal for psychosocial interventions (12–14). In terms of support, engaging activities and caregiving focusing on interactive capabilities of PwD provide essential ways to enhance social connections (15–17). Research indicates that positive communications that promote meaningful engagement may reduce anxiety, depression and social isolation in PwD while promoting connectedness and well-being (18, 19). This argues for increased focus on activities that enrich the caregiving dyad and enhance the positive aspects of the caregiving relationship when coping with a disease or illness. Social interventions and support services targeting social participation for both PwD and their informal caregivers have the potential to help the caregiving dyad to adapt and live well with the condition (15, 16, 20). Considering that the opportunities to participate in social activities for caregiving dyads are greatly limited during the COVID-19 pandemic, social technology should be explored for its potential to enhance social health and dyadic relationships in a safe environment. According to one definition, social technology can be understood as “any technology that facilitates social interactions and influences social processes between people” [(21), p. 3]. Technology-based interventions have shown promise in engaging PwD in meaningful activities, with positive impacts for interaction and social participation (20, 22). Informal caregivers reported that a convenient tablet technology provided more enjoyable and meaningful interaction with their relative, which supported their relationship (23, 24). Where social technology stimulated joint activity for the dyad, this in turn contributed to increased communicative interaction by providing a conversational focal point where they could share experiences (25, 26). Given that sufficient user support is provided, the potential of technology supporting dyadic interaction that actively engages the PwD and their caregiver in meaningful social activities can be realized (22).

These aforementioned outcomes are the overarching goals of I-CARE, an intervention study involving a tablet-based activation system specifically designed to actively engage PwD in social interactions (27, 28). In an ongoing feasibility study of I-CARE, the impact of this system on social health, dyadic relationships and caregiver burden is being evaluated among community-dwelling dementia caregiving dyads. However, it can be expected that, given COVID-19's extensive impact on society, it will also influence any social intervention undertaken during and after the pandemic. There is reason to believe that community-dwelling dementia caregiving dyads have been impacted by the ongoing pandemic and the resulting activity restrictions, but little research exists on this topic thus far. Furthermore, it is crucial to understand the contextual factors that might influence any intervention (e.g., I-CARE) in a specific setting (29). Therefore, as part of the ongoing I-CARE intervention, this paper reports on the experiences of three participating caregiving dyads in the context of the ongoing COVID-19 restrictions.

### Objectives and Aim

The aim of this study is to assess how COVID-19 has impacted the social participation and dyadic interactions of community-dwelling dyads in a dementia caregiving context. In addition, the study explores how the dyads use social technology in everyday life, and their motivation for participating in the I-CARE social technology intervention (described further below). Specifically, this study is guided by the following research questions:

1. How have COVID-19 restrictions impacted social and leisure activities for community-dwelling dementia caregiving dyads?
2. How has social technology contributed to mitigating the impact of social isolation and limited support offers available to caregiving dyads?
3. What barriers and facilitators exist in using social technology among caregiving dyads in the context of living with dementia?

The findings from this small pilot study will enhance communication and spark a discussion about this important topic of how the COVID-19 pandemic has impacted social participation and dyadic interactions among PwD and their caregivers.

## Methods

### Study Design and Participants

This pilot case study is based on interview data from three dementia caregiving dyads (i.e., a PwD and his/her informal caregiver). The data were gathered as part of an ongoing feasibility trial of the I-CARE intervention. In the intervention, PwD and their informal caregiver engage in social activities together, supported by I-CARE. The tablet-based activation system provides user-specific content specifically designed for PwD by tailoring the activities to the individual activation needs and capabilities of PwD. These activities include image galleries, music, videos, games, quizzes, proverbs and stories. The I-CARE system is described further elsewhere (27, 28).

From December 2020 to March 2021, caregiving dyads were recruited to “pilot”-I-CARE through Day Centers, support groups, and the Dementia Information- and Coordination Center (DIKS), all located in Bremen, Germany. These collaborating partners reached out to clients they deemed eligible and potentially interested in participating in the I-CARE intervention and provided them with the contact information of the I-CARE research team so that interested caregiving dyads could directly contact the researchers. Eligible dyads were living at home, with the informal caregiver either cohabiting with the PwD or visiting at least twice per week (on average). People with a formally confirmed diagnosis of dementia were included, regardless of the type or stage. Participants were excluded from the study for the following reasons: participating in another intervention trial, bedridden/heavily immobilized, deafness/severe hearing impairment, blindness/severe visual impairment, or diagnosed with schizophrenia or substance addiction.

Dyads who expressed interest in participating received comprehensive written and verbal information about the goals of the I-CARE intervention, including eligibility criteria, the nature of the intervention, data collection procedures, and a written consent form. Caregiving dyads who, after considering the study, agreed to participate and signed the informed consent form—were scheduled for baseline measurements, during which they also received a tablet with the I-CARE system pre-installed, designed to actively engage them (caregiver dyad) in social interactions (27, 28).

The Deutsche Gesellschaft für Pflegewissenschaft e.V (DGP) approved the study, and informed consent was obtained from all dyad members. This study is based on interview data from the baseline data collection of three caregiving dyads, for whom data was available (i.e., more dyads had yet to be recruited). This study is designed as a case study (30) nested within the ongoing I-CARE intervention. A case study design is advantageous, as it allows close exploration and inspection of each caregiving dyad's experience during the ongoing COVID-19 pandemic.

### Data Collection

Quantitative measurements were collected to assess the effect of the trialed technology, which are part of the ongoing intervention and will be reported in a future publication. Participant characteristics were also collected, including age, gender, number of children, and number of caregiving hours provided by the informal caregiver. In addition to documenting the type of dementia, the severity of functional disability associated with cognitive impairment was assessed using the Functional Assessment Staging questionnaire [FAST, (31)]. This is a functional scale designed to allow both professionals and caregivers to chart the decline of people diagnosed with dementia in seven dementia-related functional disability stages, ranging from one (no cognitive deficit) to seven (severe). For each charted stage, clinical descriptions of common abilities are assessed, such as memory capabilities, personal hygiene, and taking care of oneself (e.g., dressing oneself or eating). This hierarchy of functions (i.e., disability stages) has been found to be related to cognitive decline within dementia. In this study, the FAST instrument was used—as proposed by its authors (31)—as a proxy rating, by the interviewed informal caregiver, of the PwD's functioning. One round of semi-structured interview was conducted with each of the three caregiving dyads at baseline before they enrolled in the I-CARE intervention. A trained research assistant with experience in qualitative and quantitative research methods conducted the interviews in German. The interviews, which were conducted in-person at the dyads' homes (practicing social distancing using protective masks), were audio recorded and lasted between 1.5 to 3 hours.

### Data Analysis

The audio-recorded interviews were transcribed verbatim and translated from German to English by a German native speaker. The transcripts were then transferred into NVivo version 12 (QSR International Pty Ltd., 2020), which facilitated a systematic organization of the data. An inductive thematic analysis approach was undertaken according to the thematic analysis guidelines described by Braun and Clarke (32). Thematic analysis of the interviews was conducted within a realist framework, reporting on the experiences, meanings, and the reality of the participants (32). The translated transcripts were read multiple times to get familiar with the data. Thereafter, the first and last authors (VH and EAA) independently coded features of the data and collated the codes and initial themes. They then discussed, reviewed, and revised the themes at the level of the individual coded extracts and the full data set by sorting the codes, transferring codes under similar sub-themes, collapsing sub-themes, or creating new overarching themes. The main themes and subthemes were refined and named collaboratively. Finally, the preliminary themes were discussed between all authors to develop and modifying them, achieving a consensus.

## Results

### Participants

As shown in Table 1, three caregiver dyads were part of this study, and in all three cases, the husband had dementia and participated in the study together with his wife. The stage of dementia among the participants was mild (“Dyad A”), moderate to severe (“Dyad B”), and severe (“Dyad C”). Carers and care recipients were, respectively, 57 to 83 and 58 to 85 years old.

**Table 1:** Participant characteristics.

**Participant characteristics**	**Dyad A**		**Dyad B**		**Dyad C**	
	**PwD**	**Carer**	**PwD**	**Carer**	**PwD**	**Carer**
Age	80	70	85	83	58	57
Gender	Male	Female	Male	Female	Male	Female
FAST^a^	2	n/a	5	n/a	7	n/a
Children^*^	Yes		No		Yes	
PwD caregiving needs	Moderate		High		High	
Supportive service(s)	Day center		Home care		None	
Using assistive technology	No		Yes		No	
Using social technology	Yes		No		Yes	

* *None of the participants had children that were minors; all had moved out by the time of the dementia diagnosis was set*.

a *FAST, Functional Assessment Scale Test*.

Dyad A had some experience with social technology, but they had no previous experience with computer tablets. Their grandchildren had equipped their home with Wi-Fi, and although they were not sure of how it functioned, they both had their own smartphones, connected to their home-internet. The husband with dementia had a mild case of the condition and attended a local Day Center three times per week, while the wife was active in dementia support groups. Both members of the caregiving dyad were relatively healthy overall and did not need assistive technology to help them perform everyday life activities.

Dyad B had the oldest participants and no prior experience with social technology other than their TV, which they watched frequently, together. They were, however, familiar with assistive technology, as they had a stairlift and a specialized bed at home to support them in living together at home. They did not own a smartphone but were enthusiastic to try the I-CARE system during the introduction session. Traveling and dancing had been a significant part of their lives together, and they had been steady dance partners for more than 40 years. Now in their current lives, they enjoyed a quiet lifestyle at home. The couple did not receive any formal support services other than having a paid assistant to help them at home, nor did they attend any support groups.

Dyad C was young, well-familiar with technology, and highly motivated to participate in this study. The dyad struggled with adjusting to the illness, which had been diagnosed only a year prior. Since then, the speaking capabilities of the husband had rapidly declined, which the wife experienced as somewhat traumatic. Nevertheless, the dyad was not receiving formal care services at the time of the baseline data collection, and they did not use assistive technology to help them at home. Although his ability to speak was limited, the husband was actively engaged during the interview and was enthusiastic in contributing to the conversation and emphasizing the statements of his wife.

### Themes

When analyzing the baseline interviews from the three cases in this study, two overarching themes were identified: “living with dementia during COVID-19” and “the role of technology in a pandemic.” These two main themes and their associated subthemes were closely connected in that they mutually influenced one another but were nevertheless sufficiently distinguishable to remain as individual themes. An overview of themes, subthemes, and codes is provided in Table 2. The dyads differed in terms of their dementia diagnosis, life situations, and experiences, and how they perceived the impact of COVID-19 on their lives. We therefore present the results (including direct quotes) case by case, organized under themes, and subthemes.

**Table 2:** Overview of themes, subthemes, and codes.

**Themes**	**Sub-themes**	**Codes**
Living with dementia during Covid-19	Social and leisure activities	Watching TV, keeping busy with chores, new inside activities, cooking, boredom, lonely island, feeling trapped at home, digital socializing insufficient
	Dyadic interactions	Lacking new input, maintaining daily routines, shared interests, dementia stage, communication difficulties
	Adjusting as caregiver	Caregiver burdens (concern for husband's health, frustration with restrictions, “moments lost,” restricted enriching activities, letting old interests go), coping strategies (adjusting the language, respite through support services, respite through friends, learning, and adapting, “it is what it is”)
The role of technology	Facilitating social activities	Source of entertainment, staying connected with friends, reminiscence
	Facilitating dementia care-related activities	Support groups, dementia information centers, speech therapist services, assistive technology
	Barriers and facilitators to utilizing technology	Tech literacy, basic experience, user willingness, knowledge of benefits, curiosity, sufficient support, clear instructions, easy language
	Underlying motivation to invite technology into dyadic interactions	Trying something new, keeping up with society, develop new skills, topic introduction, conversation support, engaging care recipient

#### Living With Dementia During COVID-19

The consequences of COVID-19 restrictions on community-dwelling dyads need to be understood in the context of living with a dementia diagnosis. The differences in the experiences reported by the dyads, and how they perceived the impact on COVID-19 on their lives could, to a certain extent, be linked to their caregiving situation—such as the stage of dementia and the availability of supportive networks. Three subthemes were identified under this theme: (i) social and leisure activities, (ii) dyadic interactions, and (iii) adjusting as a caregiver.

##### Social and Leisure Activities

When looking into how the COVID-19 restrictions had impacted social participation among the participants, it quickly became evident that the dyads' perceptions of how COVID-19 restrictions had impacted their social participation was dependent on how active the dyads were within their social networks before the outbreak and whether they used remote alternatives to maintain their network during the pandemic.

For Dyad A, the restrictions had created a lonely island where the dyad mainly was isolated. The couple expressed that they needed their wider social network and found it discouraging not to have access to their social circles. Although the dyad used technology to a certain extent for social purposes, they perceived it to be an inadequate substitute for in-person contact. Therefore, rather than the remote presence of their network being a supportive resource, the dyad's restricted access to it was a source of anger and sadness. “*I have the feeling we live on an island alone and there is contact but only by smartphone and [searching for words] e-mail and telephone but friendships… you kind of feel that everything is somehow different.” (Wife, Dyad A)*. As the couple expressed, the long duration of the COVID-19 restrictions and the lack of adherence to the rules by some people in the community were immensely frustrating. The isolation was not only a major burden for the wife, but she was also concerned about who would take care of her husband if she contracted the virus or got sick.

Dyad B lived a more relaxed lifestyle and did not feel that the COVID-19 restrictions had particularly limited their leisure activities. The husband had mobility issues, which necessitated following placid everyday routines. They had shared interests in activities they enjoyed doing at home. As a result of the outbreak, they had invested in a board game to keep them occupied. *Interviewer: “A typical day together. How would you describe it?” Carer: “Very cozy. Calm breakfast in the morning…then at 11am an assistant comes around… and yeah, the day is being enjoyed fully relaxed, lunch, drinking coffee, in the evening snugly looking into the distance and that's it.” (Wife, Dyad B)*.

Dyad C had a more active lifestyle than the two other dyads, despite the husbands' severe stage of dementia. Before the imposed restrictions, they frequently went to the city to enjoy their evenings at restaurants, the cinema, or visiting their friends. They thus felt that there were not enough activities available to engage in while at home during the community lockdown. Even so, and due to their young age and familiarity with technology, they kept in touch with their social network through videoconferencing. Therefore, they did not perceive their social network to be severely limited by COVID-19 restrictions. “*I cooked a lot, [and] that was when I noticed how often we eat out. Never tried so many recipes and sports I'm doing outside anyway so nothing changed in that sense. And we still met with other people or used Zoom to keep this up.”* (Wife, Dyad C).

##### Dyadic Interaction

Overall, none of the dyads reported that their dyadic interactions had been influenced by the ongoing pandemic. Their “togetherness” was upheld by maintaining their daily routines and adjusting them as needed, given the ongoing restrictions. Although the couples' interactions with their partners remained as before the outbreak, the framework within which the dyads communicated with one had changed due to COVID-19 restrictions, which had some implications for how the dyads coped with being isolated together.

Dyad A described how they were mutually dependent on each other to keep busy at home and being each other's conversation partner. Although they felt that their dyadic interactions had not significantly changed since the COVID-19 restrictions were imposed, the lack of variation in other conversation partners took its toll. The husband had frequent interactions with his wider social network through his Day Center activities. Although fewer participants were allowed to attend the Day Center, the husband enjoyed the time he spent and the activities he engaged in with his comrades. His wife, on the other hand, had no in-person contact with her own social network, but she appreciated the respite the Day Center provided. This asymmetrical access to their wider social network seemed to affect how the couple dealt with their isolation: “*We are both kind of dependent on each other and that is… it's been going on for a long time already and that does make me sad. I don't know how my husband feels but I think he is a bit calmer about this*.” (Wife, Dyad A).

Dyad B was the oldest, and they had no children or regular contact with a wider social network. Consequently, they did not perceive the COVID-19 restrictions to have impacted their dyadic interaction or their need for interactions outside of their relationship. Additionally, they did not feel that the diagnosis of dementia had so far impacted their dyadic interactions. They shared similar interests in conversation topics and TV shows, and therefore enjoyed their conversations and time together. “*We go about like we've always done.”* (Wife, Dyad B).

Although Dyad C shared many interests and maintained an active lifestyle together, they experienced a heavy strain on their dyadic interaction with one another since the dementia onset. They felt the COVID-19 restrictions had not changed the framework within which they interacted, but their conversations were severely limited due to the dementia diagnosis. Their shared interests in TV shows and doing sports helped them interact non-verbally, but the wife felt a major burden in upholding their conversations. “*There's barely dialogue, none happening. So, I try asking him stuff… and I feel it does get to him, but I barely get answers, short two, three words. Well, sometimes it's a bit better, but there's barely any exchange possible*.” (Wife, Dyad C).

##### Adjusting as Caregiver

The wife in each dyad was still adjusting to the caregiver role, the emerging burdens that the dementia diagnosis entailed, and developing coping strategies. Then with the COVID-19 outbreak in March 2020, they had to further adjust to the role in the context of the pandemic, taking on an even larger responsibility for their spouse and their own psychosocial well-being.

The wife in Dyad A had learned how to be more objective in her reactions to her husband's dementia trajectory. She learned to accept that dementia could affect couples in their age group, and this enabled her to be less frustrated in their dyadic interactions. She had participated in support groups before COVID-19, which had been crucial in helping her cope and adjust to the diagnosis. “*And I still talk about this with my husband because I also notice, he can still understand this but…but he partially can't implement them. Yeah, and sometimes I ask myself ‘Am I doing it right or am I doing it wrong?' That was very bad in the beginning, since then I got some kind of confidence in this […] a lot of things I didn't have to do in the past, I have to do now. My life has become full because of this sickness and…yeah. What am I supposed to say more about this? It is what it is*.” (Wife, Dyad A). However, in-person dementia support groups were mostly unavailable due to the pandemic. Even so, the wife sought out the services remotely (via phone, e-mail) for emotional support; but she did not have access to any videoconferencing tools.

As a part of adjusting to a life with dementia, Dyad B received support from a speech therapist to help maintain the husband's communication abilities. They also had a home assistant to help them in everyday life activities, primarily to give the wife respite. The wife expressed that she did not get much time to herself due to her husband's physical condition. Instead, she felt that she needed to be available at home. Their previously active lifestyle as dance partners was no longer possible due to her husband's illness, and she had stopped attending her fitness studio to care for him. Considering that her activities and social participation were already limited prior to the pandemic, the community lockdown fit with her present lifestyle and actually offered some solace. “*Yes… I do stuff at home… [searching for words] I was in the fitness gym for 16 years, but I can't do that anymore because I don't want to leave him at home for that long. And it's closed right now anyway*.” (Wife, Dyad B). She had not sought out any support groups for herself and expressed little interest in doing so. The couple considered themselves sociable, but they did not express any frustration in being isolated from their social network, which they did not describe as a source of support. Therefore, they felt that the pandemic had not impacted their adjusting to the dementia diagnosis.

Dyad C had received the dementia diagnosis one month before COVID-19 was declared a global pandemic and thus had little time in adjusting as a caregiver before the lockdown. This situation greatly affected how the dyad was adjusting and the wife's coping strategies. Despite the husband's rapidly declining health, the dyad received no formal support in their everyday life activities. However, they did have a speech therapist who carried out regular exercises with the husband in-person or via videoconference. The wife had sought out several support groups shortly after the diagnosis, and while some of them remained active during the pandemic via virtual platforms, she expressed being frustrated that not all of the support groups continued in this manner. The couple was relatively young, and the wife felt that the older adults in the support groups, unlike her, did not see the benefits of virtual meetings. She perceived this to be the main reason for the limited support groups available during the pandemic. “*I had begun searching for support groups and then when I found one it only took place once and then the lockdown came and because there are much more older people than me, who don't want to use Zoom, that wouldn't be a problem for me at all*.” (Wife, Dyad C). Compared to Dyads A and B, the informal support network was the primary source of support for adjusting to the diagnosis for Dyad C. They had friends available to them in-person or through videoconferences such that they managed to maintain their social network despite the diagnosis and the ongoing pandemic. Furthermore, the support they received from friends was a vital source of respite for the wife, allowing her time to stay occupationally active and maintain her active lifestyle.

#### The Role of Technology in a Pandemic

The role of technology in promoting active participation in daily life was an inherent part of the discussions, given that the interviews were part of the baseline assessments in a technology-based intervention. The subthemes included (i) *facilitating social activities*, (ii) facilitating *dementia care-related activities*, (iii) *barriers and facilitators to using social technology*, and (iv) *the underlying motivation to invite technology into dyadic interactions*.

##### Facilitating Social Activities

TV was the main source of all the dyads' shared leisure activities. While this was part of the usual routine of Dyad B before the pandemic, TV had substituted many of the more active routines that Dyads A and C shared before the pandemic. The wife in Dyad A expressed anger in how this had become their new routine and longed to take up their previous activities of going out and having visitors. “*In the evening we watch TV, news and then I just relax in front of the TV and I sometimes I get angry. I used to get a lot of stuff done in the evening but that is no longer the case, and yes the day is as we do it every day, if we are alone, if we can't get any visitors, if we can't go anywhere*.” (Wife, Dyad A). Dyad C shared the same views but expressed more patience about waiting for the social activities to become available again. Dyad C was proficient in online social platforms and videoconference tools and could still virtually partake in social activities. For Dyad A, technology, besides serving as a tool to reach *out* to their networks by telephones and e-mails, also served an important function *within* the dyad. Having traveled together in the past, the husband had digitalized the photos from their trips onto DVDs. The dyad frequently viewed and reminisced over these picture slide shows together, bringing joy to them.

##### Facilitating Dementia Care-Related Activities

Regarding dementia care-related activities, a distinction emerged between technology for supporting social vs. physical needs. Dyad B was dependent on assistive technology to support dementia care-related activities. They had a stairlift and a sickbed that enabled them to live independently at home. “*Without a stairlift it wouldn't be possible [to live here]. It's superb*!” (Wife, Dyad B). Considering that the wife showed little interest in participating in dementia support groups, no technical tools to facilitate social support were used.

Dyad A and C utilized no assistive technology to support them at home, but rather, they used technology as a tool for receiving social and emotional support in coping with the condition. While the wife in Dyad A used simpler technology such as telephones and e-mail to reach out to her support network, the wife in Dyad C also participated in larger support groups through videoconferences. This was also Dyad C's primary tool for receiving support from their speech therapist.

##### Barriers and Facilitators to Using Social Technology

Several barriers and facilitators to using social technology were found, related to *tech literacy, user willingness*, and *sufficient support*. These are interrelated and are therefore not presented as individual subthemes, but rather as the most important contextual factors expressed by the three dyads.

###### Tech Literacy.

It was clear that basic knowledge about available social technology and its function was the most essential facilitator for its uptake. Dyad A and C's enjoyment in using social technology in their shared activities or connecting with their network stood in clear contrast to Dyad B's. The latter had little experience with social technology.

###### User Willingness.

The issue of limited tech literacy was voiced by the wife in Dyad C, who meant that this also influenced the willingness of older adults who did not see the benefit of new technology to try them out. “*But the problem is that the older ones… well the technology… they don't get it, but I think it's better than nothing*” (Wife, Dyad C). Yet, the unfamiliarity of Dyad B with social technology beyond their TV did not reflect their *willingness* to try it. The wife was curious and enthusiastic to try out new technology and thereby keep up with the development. This, however, depended on her having appropriate tech support.

###### Sufficient Support.

The insecurities that older adults might feel in using new technologies were also visible in Dyad A. Although the husband enjoyed using his technological devices, his wife expressed some discomfort and insecurity when she did not know how to handle new technology. The wife emphasized that in order to realize the benefits of a device, she first needed to have confidence in using it. Dyad B expressed that as long as sufficient support was available, they would not have any reservations in learning how to use new technology. However, the main problem was that they did not know how to educate themselves about these devices, and COVID-19 restrictions limited access to the available support in doing so. The dyad also emphasized that proper support includes appropriate language adapted to the needs of the respective users in user-manuals. “*[…] if the manual is well written… sometimes it is bad […] a lot of things imply that a certain standard [of knowledge] exists, but for laypeople this is not good*.” (Wife, Dyad B).

##### The Underlying Motivation to Invite Technology Into Interactions

The dyads' underlying motivation to try I-CARE complemented their reflections around the main barriers and facilitators for using social technology. Each dyad's (case) motivations are presented below.

Dyad A saw I-CARE as an opportunity to try something together as a couple, for they already had such great experiences with their digital reminiscence sessions together. I-CARE was, therefore, another activity that could enable them to engage in something new. “*For me, I feel I can do something together with my husband, try something out. Both of us. And that would be nice. [turns to husband] What about you?*” (Wife, Dyad A).

“*I have three laptops and I wouldn't want to miss them. In a way, this is basically the fourth one, and it can't hurt. It's fun. I enjoy watching the pictures I took during my travels. A lot of stuff you forget, right? And I find that fun. If you can't be outside so much anymore*.” (Husband, Dyad A).

Dyad B was also curious to try something new even though they had little to no experience with social technology except their TV. They saw I-CARE as an opportunity to keep up with societal progress and evolve their understanding of technology in general. “*Well yeah. This excites me more and more… also having a tablet… and you have to go along with the time.”* (Wife, Dyad B).

The wife in Dyad C had a very specific goal in mind. She hoped that the technology, namely using I-CARE with her husband, could facilitate better conversations between them, and reduce the pressure she felt in initiating and maintaining their conversations. “*I hope from this [sic] that we can exchange conversations a bit better, that more comes from my husband.”* (Wife, Dyad C).

## Discussion

This qualitative study is part of an ongoing feasibility study of the I-CARE social technology intervention—whose effects on dyadic relationships and their interactions, caregiver burden, and quality of life will be evaluated in a future study. This pilot study aimed to promote discussion on exploring: how COVID-19 restrictions have impacted dementia caregiving dyads in terms of their social and leisure activities, how social technology can mitigate the isolation they currently experience, and what facilitators and barriers exist for this population in using social technology. We identified two overarching themes in our analyses: (i) *living with dementia during COVID-19 (subthemes: social and leisure activities, dyadic interactions, adjusting as caregiver)*; and (ii) *the role of technology in a pandemic (subthemes: facilitating social activities, facilitating dementia care-related activities, barriers and facilitators to using social technology, the underlying motivation to invite technology into interactions)*.

The extent to which dementia caregiving dyads had to adjust to the COVID-19 restrictions seemed to be influenced by two main factors: how socially active the dyad had been within their social support networks, and their familiarity with social technology. Dyad C, who had lived a socially and physically active life prior to the outbreak, still heavily relied on their network for support, which they maintained largely through social technology. Therefore, despite having received the dementia diagnosis shortly before COVID-19 was declared a global pandemic, they coped well with the restrictions and the limited activities available outside the house. On the other hand, Dyad A, whose lives were also rather social before the outbreak, experienced the isolation as frustrating and missed their social network. Their limited knowledge of social technology meant that they had to maintain their social contacts via telephone or e-mail. However, this form of contact did not sufficiently mitigate their isolation, which the wife found especially challenging. This is consistent with research showing social participation as a key determinant of healthy aging (5, 33, 34). Contrary to these findings, Dyad B (who also had limited knowledge of social technology) did not feel that the COVID-19 restrictions had impacted their lifestyle in a noteworthy matter. They had stayed mostly at home with limited contact with a wider social network. Therefore, they did not perceive the restrictions imposed following the COVID-19 outbreak as burdensome.

The findings indicate that the availability of the supportive networks was closely intertwined with the wives' strategies in adjusting and coping as caregivers. Dyad C's proficiency with technology was an important factor in receiving support from their informal network, on which they were highly dependent. Therefore, the wife found the unwillingness among the older adults to utilize technology frustrating because it limited the available opportunities for support (e.g., group meetings could not be held remotely). However, the willingness and curiosity in Dyad B (the oldest) to try new technology stands as a contrast to the perception that older adults are disinclined to new technology, and contributes to the increasing evidence debunking the old misconception of older adults being unable or unwilling to follow the technological development (35–38). Dyad B was enthusiastic about trying out the I-CARE system and wanted to acquire their own smartphones. They were, in other words, willing to try technology new to them, but this was dependent on having appropriate assistance. Therefore, it seems that the significantly lower adoption of new technology among older adults compared to younger generations (39) is not necessarily grounded in technology aversion (40), but rather the lack of sufficient support in doing so. If we are to encourage PwD and their caregivers to engage with new technology, we need to dismiss the stereotype of older adults as “technophobes” (41) and instead direct our attention on how to sufficiently support this population in doing so. For instance, the wife in Dyad A did not know how to use videoconferencing tools and therefore did not see the opportunities technology offered for facilitating social participation. One might argue that had she been supported to interact with her support group or family over videoconferencing platforms, this might have felt closer to in-person social participation than what phones and e-mails could provide. Thus, our findings and existing literature suggest that social technology, which has the potential to mitigate some of the isolation that has shaped our society since March 2020, is inaccessible to those who might need it the most.

Another clear distinction between the youngest and oldest dyads was the extent to which they were dependent on their informal social network. Contrary to Dyad A and B, Dyad C relied on their friends rather than formal services for support. These differences can partly be explained by the fact that they received the dementia diagnosis shortly before the outbreak, giving the dyad little time to investigate what formal support offers existed. Dyad C reported receiving support from their friends, a finding that might also be explained by their young age and the likelihood that their informal network mostly consisted of healthy individuals who were also familiar with social technology. This is supported by findings from research implying that the successful uptake of technology is dependent on becoming a part of the social network and daily living of PwD (42), which seems to be the reason Dyad C was able to stay socially active while in-person contact was impossible. Conversely, Dyad A was dependent on the local Day Center for in-person social interaction for the husband. This was not possible for his wife, who expressed that she had a harder time coping with the isolation than her husband. This asymmetrical access to the wider social network in the dyad suggests that it is also crucial to maintain support groups for caregivers during crises like this pandemic, as research indicates that social support is an imperative source for caregiver resilience (8, 43).

It was clear that the social network was important to help the wife in Dyad C cope, while the wife in Dyad A found the sudden lack of her social network a major source of sadness and frustration. Dyad B, however, expressed that they did not feel that the pandemic had impacted their lives much, not in terms of available support or their dyadic interaction. Perhaps the wife would have been interested in participating in *virtual* support groups if she had participated in such before the outbreak. COVID-19 did not influence the availability of home-assistant services or assistive technology for Dyad B, which might also explain why they did not feel very influenced by the COVID-19 restrictions. The husband in Dyad B had physical rather than cognitive ailments, which might explain why the diagnosis had such a low impact on their conversations. Contrarily, Dyad C struggled greatly in communicating with one another, which is consistent with studies that suggest that the deterioration in cognition and communication in dementia contribute substantially to the experienced burden among caregivers (44, 45). Due to the nature and stage of the husband's diagnosis, the wife in Dyad C experienced challenges upholding their conversations and understanding how to adapt her communication. This also explains why Dyad C had different underlying motivations in inviting technology into their dyadic interactions. While Dyad A and B wanted to try a new activity together, the wife in Dyad C saw I-CARE as an opportunity to help the couple communicate better. Research suggests that using technology to support interactions can help reduce the pressure on the conversational partner by keeping the interactions more symmetrical (22, 46), as well as help caregivers better understand the PwD (22). A technology-based conversation aid similar to I-CARE was reported to have beneficial effects on social interaction, quality of life and dyadic relationships (46–48).

One thing all three dyads had in common was using the TV as a social activity together while other activities were restricted. However, attitudes toward this activity differed between the dyads. For Dyad B, who had already enjoyed a relatively inactive lifestyle before the outbreak, watching shows together was a sufficiently engaging activity for the dyad to do together. However, watching TV is a relatively passive activity that does not substitute social participation in the wider social network—with social activities and positive interactions being crucial for the well-being of caregiving dyads and maintenance of cognitive function as one ages (47–49). The wife in Dyad A expressed anger in how most of their evenings were spent in front of the TV instead of going out or having visitors. Nevertheless, their TV also enabled reminiscence activities for Dyad A, which they both enjoyed doing together. Their positive experiences with digital reminiscence might explain some of the underlying motivations the couple had in inviting technology into their dyadic interactions. Furthermore, the husband in Dyad A owned several personal computers, with which he frequently interacted. Similarly, the husband in Dyad C found much enjoyment in a computer course he had participated in after being diagnosed. This is in agreement with research that indicates positive effects of computerized training programs on social participation among people with cognitive impairments (50, 51). It may seem that having basic experiences with technology is an extensive facilitator to encourage older adults to try *new* technology, such as social technology, and thus benefit from what such technology offers. Realizing these benefits calls for spearheaded efforts to empower older adults to become familiar with, see the benefits of, and use social technology.

### Limitations

The purpose of this pilot study is not to generate results that can be generalized to the larger population of PwD and their caregivers, but rather to provide contextualized details to support transferability to other contexts or settings. The three couples represent the most common typology of dementia caregiving dyads (couple relationship, the wife being the caregiver) (52). The case studies provide preliminary insights into how the ongoing pandemic has shaped and influenced the lives of dementia caregiving dyads—a population group that is largely dependent on support from their social network and the availability of activities outside their homes, to uphold social participation. Our findings are possibly influenced by recall bias, considering that the interviews were conducted almost one year after COVID-19 was declared a pandemic. For example, having acclimated to COVID-19 restrictions over time, participants might have underreported the magnitude of the pandemic's adverse effects, especially during the first wave. In the three dyads, the husbands had dementia, which influenced the extent to which they were able to provide comprehensive descriptions of their own experiences and reflections since the COVID-19 outbreak. The wives, therefore, provided most of the information during the interviews, but both the interviewer and the spouse regularly asked if the husband agreed or wanted to make further comments to the discussion. Supplementing audio recordings and field notes with video observations in the moment of storytelling might be a viable strategy to accommodate the person with dementia to express their thoughts and experiences in cases where the stage of dementia makes it difficult to actively participate in interviews. Finally, to adhere to COVID-19 restrictions, the interviews were conducted following social distancing guidelines including protective masks. A desire to limit interviewers' and interviewees' exposure to one another might have contributed to shortening the length of the interviews, thereby reducing the richness of data gathered.

## Conclusions

This study contributes to the growing research on how COVID-19 has influenced PwD and their caregivers (2, 4, 53–55). The effects of discontinued health and social services such as support groups and respite services on the social health of community-dwelling dementia caregiving dyads through the pandemic require further investigation. Given the concerns about potential adverse impacts of the pandemic on dementia caregiving dyads, future research should examine possible long-term effects of COVID-19, such as increased mortality, morbidity, or institutionalizations. The dyads differed in how COVID-19 restrictions impacted their lives and how they coped with dementia, which revealed their different motivations for wanting to invite technology into their social interactions. The influence of demographic characteristics in how dyads adapt to a life with dementia should be further examined, in terms of involving their social network and the dyads' prerequisites for using social technology to do so. Special attention should be given to characteristics such as age (e.g., the experiences of younger vs. older dyads), dementia stage, and even previous experience with social technology. Video observations and other appropriate and innovative ways to meaningfully engage PwD in research should also be incorporated in future studies. During and beyond this pandemic, social technology can be a valuable addition to promote social participation in this population, especially when in-person social contact is very limited or not possible. Successful uptake of social technology is dependent on customizing it to the individual's needs and conditions. Efforts are therefore needed to tackle barriers that exists for older adults with or without dementia in using such technology.

## Data Availability Statement

The raw data supporting the conclusions of this article will be made available by the authors, without undue reservation.

## Ethics Statement

The study was were reviewed and approved by Deutsche Gesellschaft für Pflegewissenschaft e.V. All participants provided their written informed consent to participate in this study.

## Author Contributions

VH was responsible for the conception and design of the study, organizing data collection, and analyzing the data (coding data, collating codes, and initial themes and collaboratively naming and refining themes and subthemes with EAA) and writing the first draft. EAA was responsible for analyzing (coding data, collating codes, and initial themes and collaboratively naming and refining themes and subthemes with VH) the data and editing the manuscript. KW-O supported in conceptualizing the study, reviewed and refined the initial themes, read, and provided substantial edits on the manuscript. All authors read and approved the final manuscript.

## Funding

This study arises from the project DISTINCT (Dementia: Intersectorial Strategy for Training and Innovation Network for Current Technology), which is funded by the Marie Skłodowska-Curie Innovative Training Networks (MSC-ITN) under the European Commission's Horizon 2020 program (Grant Agreement No. 813196). The study is also partially funded by the Leibniz ScienceCampus Bremen Digital Public Health (lsc-diph.de), which is jointly funded by the Leibniz Association (W4/2018), the Federal State of Bremen and the Leibniz Institute for Prevention Research and Epidemiology—BIPS. None of the funding networks were actively involved in the design of the study, the data collection, the analysis, the interpretation of the data, or in writing the manuscript.

## Conflict of Interest

The authors declare that the research was conducted in the absence of any commercial or financial relationships that could be construed as a potential conflict of interest. The handling editor declared a past collaboration with one of the authors KW-O.

## Publisher's Note

All claims expressed in this article are solely those of the authors and do not necessarily represent those of their affiliated organizations, or those of the publisher, the editors and the reviewers. Any product that may be evaluated in this article, or claim that may be made by its manufacturer, is not guaranteed or endorsed by the publisher.

## Abbreviations

DGP: Deutsche Gesellschaft für Pflegewissenschaft e.V
DIKS: Dementia Information- and Coordination Center
DISTINCT: Dementia: Intersectorial Strategy for Training and Innovation Network for Current Technology
FAST: Functional Assessment Scale Test
MSC-ITN: Marie Skłodowska-Curie Innovative Training Networks
PwD: People with dementia.
